# Oral microflora in preschool children attending a fluoride varnish program: a cross-sectional study

**DOI:** 10.1186/s12903-016-0325-6

**Published:** 2016-12-08

**Authors:** Maria Anderson, Margaret Grindefjord, Göran Dahllöf, Gunnar Dahlén, Svante Twetman

**Affiliations:** 1 Division of Pediatric Dentistry, Department of Dental Medicine, Karolinska Institutet, Huddinge, Sweden; 2 Department of Pediatric Dentistry, Eastman Institute, Public Dental Service, Stockholm, Sweden; 3 Department of Oral Microbiology and Immunology, Sahlgrenska Academy at the University of Gothenburg, Gothenburg, Sweden; 4 Department of Odontology, Faculty of Health and Medical Sciences, University of Copenhagen, Copenhagen, Denmark; 5 Pedodonti, Folktandvården Eastmaninstitutet, Dalagatan 11, SE-11324 Stockholm, Sweden

**Keywords:** Bacteria, Caries prevention, Community health program, Fluoride varnish, Preschool children

## Abstract

**Background:**

To compare the oral microflora in preschool children attending a fluoride varnish program with a reference group receiving a standard oral health program without fluoride varnish applications. A second aim was to relate the microbial composition to the caries prevalence.

**Methods:**

Five hundred seven 3-year-old children were enrolled from a cohort of 3403 preschool children taking part in a community based oral health project. Two hundred sixty-three of them had attended caries-preventive program with semi-annual applications of a fluoride varnish since the age of 1 year (test group) while 237 had received standard preventive care (reference group). Oral samples were collected with a sterile swab and analysed with checkerboard DNA-DNA hybridization using 12 pre-determined bacterial probes. Caries and background data were collected from clinical examinations and questionnaires.

**Results:**

Gram-positive streptococci (*S. intermedius*, *S. salivarius*, *S. oralis*) were most frequently detected and displayed the highest counts in both groups. There were no significant differences between the groups concerning prevalence of any of the selected bacterial strains except for *S. oralis* that occurred less frequently in the reference group. In children with caries, *V. parvula* were significantly more common (*p* < 0.05) while strains of Lactobacillus, Bifidobacterium and Neisseria were more prevalent among the caries-free children (*p* < 0.05).

**Conclusions:**

A 2-year community program with semi-annual fluoride varnish applications did not seem to significantly influence the oral microflora in preschool children.

**Trial registration:**

www.controlled-trials.com (ISRCTN35086887) 20131216 ‘retrospectively registered’.

## Background

Early childhood caries (ECC) is a public health problem associated with impaired oral health-related quality of life and high costs for families as well as for society. There are numerous biological, medical, behavioural, psychological, cultural, and life-style factors associated with the disease but in spite of extensive research over the years, there are knowledge gaps concerning its prevention and management [1]. Fluoridated toothpaste and fluoride varnish are currently the most effective strategies to prevent ECC [2]. Therefore, such community oral health programs have been launched for vulnerable children with increased caries risk [3–6]. Little is however known on the long-term impact of such fluoride-based programs on oral bacteria. It is generally thought that a low-fluoride exposure can interact with the consecutive cycles of de- and remineralization while higher fluoride concentrations may hamper bacterial acid production [7, 8]. A reduced pH-drop would favour the growth of health-associated bacteria that prefer pH values around neutrality rather than the acid-producing and acid-tolerating species associated with caries [9]. Fluoride varnish contains typically 5% sodium fluoride (22,600 ppm F) and after application, fluoride is slowly released to the oral biofilm over a period of several weeks. Since it is not fully clear whether or not this sustained fluoride release has an influence on the bacterial composition of biofilm, it was thought of interest to apply this research question in a current community oral health project [10]. The primary aim of the present study was to compare the oral microflora in preschool children participating in an extended preventive program with semi-annual fluoride varnish applications with children receiving a standard program with no varnish applications. The null hypothesis was that no differences would be obtained between the groups. A secondary aim was to investigate the microbial composition in children who had developed early childhood caries at the age of 3 years versus those that remained caries free.

## Methods

This cross-sectional study was carried out as a part of a cluster-randomized controlled field trial aiming to evaluate the effectiveness of fluoride varnish applications on caries development in toddlers living in multicultural areas in greater Stockholm, Sweden [10]. The original trial was designed with two parallel arms that compared children receiving a standard oral health program (reference group) with children who had the same standard oral health program supplemented with semi-annual fluoride varnish applications (test group). The study comprised 23 public dental clinics involving 3403 toddlers and the intervention was carried out between 1- and 3-years of age. From the main study, 7 public dental clinics representing both programs were selected by convenience and invited to the present investigation. 507 children (263 from the test group and 244 from the reference group) were consecutively enrolled in connection with the scheduled 36-month examination. All parents gave their written informed consent after verbal and written information and an interpreter was used when necessary. The project was approved by the Regional Ethic Committee (EPN; no 2013/143–32).

### Intervention

The children in the test group received topical applications of fluoride varnish (Duraphat®, 22.5 mg of fluoride per ml, Colgate-Palmolive) every 6th month from 1 year of age (totally 5 applications) as a supplement to a standard oral health program. The standard program (reference group) consisted of yearly dental examinations with tooth-brushing instructions, fluoride toothpaste enforcement and dietary counselling. The children of the reference group did not receive any fluoride varnish applications. At the end of each dental visit, all participating children were given a free tube of fluoride toothpaste (1100 ppm) and a soft toothbrush.

### Sample collection

The samples were collected with aid of a sterile swab that was rotated inside the lips to capture saliva and supra-gingival plaque. The swab was then transferred to a test tube coded with a unique number and immediately sent by surface mail to the laboratory. The samples were stored frozen at − 20 °C until further processing. The samplings were performed between March and November 2013 and the samples were analysed within 3–15 months (median 12 months) after collection.

### Microbial analyses

The samples were processed with the checkerboard DNA-DNA hybridization technology as described by Wall-Manning et al. (2002) [11]. DNA was extracted with mutanolysin and lysozyme as previously described [12] and the DNA quality was evaluated from the UV extinction at 260 nm using NanoDrop 2000 (Thermo Scientific, Fisher Scientific, Gothenburg, Sweden). Whole genomic DNA probes were prepared from a panel of 12 bacterial species with strain designation according to the Culture Collection, University of Gothenburg (CCUG), or Oral Microbiology, Gothenburg, Sweden (OMGS): *Actinomyces odontolyticus* OMGS G67; *Bifidobacterium dentium* OMGS G174; *Capnocytophaga ochracea* OMGS 1233; *Haemophilus parainfluenzae* CCUG 12836; *Lactobacillus casei* OMGS 3184; *Lactobacillus salivarius* OMGS 3830; *Neisseria subflava* CCUG 23930; *Streptococcus intermedius* CCUG 17827; *Streptococcus oralis* OMGS 2470; *Streptococcus mutans* OMGS 2482; *Streptococcus salivarius* OMGS 2473; *Veillonella parvula* OMGS G186. The detection level was >10^4^ cells per mL sample.

### Caries data

Caries data was extracted from the clinical examination at 3 years of age performed by the child’s regular dental team as previously described [10]. The International Caries Detection and Assessment System (ICDAS II) was used according to Ismail and co-workers [13]. Furthermore, data from a structured parental interview concerning socioeconomic conditions, the child’s general health and oral health related habits were registered.

### Dropouts

Data from seven children were lost due to technical errors and these children were excluded from the final material.

### Statistical analysis

All data was processed with the IBM SPSS software (version 22.0, Chicago, IL USA). Differences in percentage distribution of bacterial growth between the groups were calculated with chi-square tests. We considered a *p*-value less than 0.05 as statistically significant. A power estimation based on the prevalence of *S. mutans* was made since systematic reviews have highlighted this bacterium as a strong biomarker for caries development in early childhood [14, 15]. We anticipated that a 50% difference in the prevalence of high counts (≥10^5^ cells) would be of “clinical importance”. With α and β set at 0.05 and 0.2 respectively, it was calculated that 170 participants in each group should be recruited to get sufficient power to limit the risk of Type I and Type II errors.

## Results

The background characteristics and caries data of the participating children is summarized in Table 1. The proportion of parents with immigrant background (other language than Swedish at home) was significantly higher in the test group (*p* < 0.05). Likewise, one or both parents were more often smoking on a daily basis in the test group. The children of the test group displayed also a higher prevalence of caries than the reference group.

**Table 1:** Study group characteristics and caries prevalence at 3 years of age

Variable	Fluoride	Standard	*P*
	(Test)	(Reference)	
	*n* = 263	*n* = 237	
Girls/boys	55/45%	53/47%	NS
Chronic disease (yes)	11%	10%	NS
Immigrant background^a^ (yes)	86%	76%	*p* < 0.05
Mother’s education (<9 years)	22%	21%	NS
Family income (<20,000 SEK/month)	46%	37%	NS
Mother and/or father smoking (yes)	42%	34%	*p* < 0.05
Candy (>1 week)	37%	32%	NS
Sweet drinks (>2 times per day)	6%	2%	*p* < 0.05
Daily tooth brushing (yes)	91%	94%	NS
ICDAS 1–6 (all lesions)	29%	17%	*p* < 0.05
ICDAS 3–6 (moderate/extensive lesions)	17%	8%	*p* < 0.05

^a^Other language than Swedish at home, *NS* not statistically significant

The microbial data is presented in Table 2. Gram positive streptococci dominated the samples in both groups; there were no significant differences in the prevalence of *S. intermedius*, *S salivarius* and *S. mutans* between the groups while *S. oralis* seemed to occur less frequently in the reference group (*p* < 0.05). Among the non-streptococci, *V. parvula*, *L. salivarius*, *B dentium* and *H. parainfluenze* were frequently harboured in both groups albeit in low counts. High counts were commonly displayed for *S. salivarius* and *N. subflava*. The results from the checkerboard hybridisation in relation to caries prevalence are shown in Table 3. The prevalence (ICDAS 1–6) for the entire study group (*n* = 500) was 23.4% and we found a more frequent occurrence of *V. parvula* among children with caries compared with those that were caries free (ICDAS 0). On the other hand, the *B. dentium*, *L. casei*, *L. salivarius* and *N. subflava* strains were more frequently detected from the caries-fee children (*p* < 0.05). No differences were obtained concerning *A. odontolyticus* or any of the streptococci strains.

**Table 2:** Prevalence (≥10^4^cells) and high counts (>10^5^cells) of selected oral bacteria in the two intervention groups

Strain	Fluoride (Test)		Standard (Reference)	
	*n* = 263		*n* = 237	
	≥10^4^	>10^5^	≥10^4^	>10^5^
*Actinomyces odontolyticus*	21	0	26	3
*Bifidobacterium dentium*	39	0	34	0
*Capnocytophaga ochracea*	2	0	4	0
*Haemophilus parainfluenzae*	38	0	33	0
*Lactobacillus casei*	3	0	4	0
*Lactobacillus salivarius*	42	2	39	3
*Neisseria subflava*	21	7	28	8
*Streptococcus intermedius*	83	7	83	5
*Streptococcus mutans*	66	4	66	4
*Streptococcus oralis*	89	3	72^a^	5
*Streptococcus salivarius*	79	15	75	11
*Veillonella parvula*	66	5	61	7

^a^Significantly lower than the test group (*p* < 0.05; chi-square test)

Values in table denote percentage

**Table 3:** Prevalence (≥10^4^ cells) and high counts (>10^5^cells) of selected oral bacteria in relation to caries

Strain	Caries (ICDAS 1–6)		Caries-free (ICDAS 0)	
	*n* = 117		*n* = 383	
	≥10^4^	>10^5^	≥10^4^	>10^5^
*Actinomyces odontolyticus*	17	2	26	2
*Bifidobacterium dentium*	23	3	40^a^	2
*Capnocytophaga ochracea*	2	0	2	0
*Haemophilus parainfluenzae*	28	2	38	1
*Lactobacillus casei*	0	0	4^a^	0
*Lactobacillus salivarius*	31	2	44^a^	3
*Neisseria subflava*	11	2	30^a^	9^a^
*Streptococcus intermedius*	80	6	84	6
*Streptococcus mutans*	63	3	70	5
*Streptococcus oralis*	75	4	82	4
*Streptococcus salivarius*	71	11	79	14
*Veillonella parvula*	56^b^	5	27	6

^a^Significantly higher compared with children with caries (*p* < 0.05; chi-square test)

^b^Significantly higher compared with caries-free children (*p* < 0.05; chi-square test)

Values in table denote percentage

## Discussion

The main finding of this study was that the oral microflora appeared basically unchanged following a supplementary semi-annual varnish program in a group of vulnerable preschool children. Thus, the null hypothesis could not be rejected. It is likely that the negative results were a consequence of, and mirrored the absent benefits of the fluoride varnish program on caries increment as reported from the main project [10] and from similar studies in young children [16, 17]. A second explanation could be that the semi-annual schedule of varnish applications was too infrequent to make a difference. It is suggested that fluoride in concentrations found in dental plaque may act as a metabolic inhibitor and lower the bacterial acid production in the oral biofilm but the clinical implications are still unclear [7]_._ Many bacteria are highly susceptible to fluoride in planktonic stages or simple biofilm models but this may not be the case in complex biofilms communities in vivo [9, 18]_._ Chau and co-workers [19] have recently shown that fluoride varnish applications can affect biofilm formation and acidogenicity but these effects were strongly reduced by time and biofilm age. Notably, the present samplings were performed approximately 6 months after the latest varnish application. A third but less plausible explanation could be that the daily exposure of fluoride from tooth paste in both groups may have obscured the results. Therefore, further clinical research to elucidate the impact of fluoride on bacterial physiology and adaption seems warranted.

Recent findings from molecular-based studies have confirmed the importance of mutans streptococci, Actinomyces and Veillonella for the development of early and severe childhood caries [20–22]_._ We were able to verify a frequent recovery of *V. parvula* but found no differences in the *S. mutans* or *A. odontolyticus* counts between caries-free children and those with initial, moderate and extensive lesions. Interestingly, we noted a higher prevalence of Lactobacillus, Neisseria and Bifidobacterium species among children free from caries. These findings support the concept that caries is more due to absence or under-abundance of beneficial bacteria rather than linked to specific pathogens [23, 24]. Thus, future research should focus on functional rather than phylogenetic diversity in order to fully understand host-microbiome interactions. An illustration of this complexity is *L. rhamnosus* that can be linked to both mineral loss [25] and caries prevention [26]. The relatively frequent detection of Neisseria and Haemophilus in our samples was likely a reflection of the low age of the present subjects and the oral sampling technique representing more oral structures than just dental plaque.

The obtained results must however be regarded with caution. First of all, the study groups constituted a convenience sample from a major project and the groups were unfortunately not fully balanced concerning socio-economy and caries. A previous study has established a relationship between social deprivation and the isolation frequencies of caries-associated microorganisms, such as lactobacilli and mutans streptococci, in 3- and 4-year-old-children [27]. However, when the data was adjusted for the imbalance between the groups concerning immigrant status, parental smoking, sweet drinks and caries, the bacterial outcome remained unchanged. Secondly, the checkerboard DNA-DNA hybridization technique has its strengths and shortcomings; the main advantage is that the method permits enumeration of large numbers of species in a large numbers of samples and hence considered as a useful tool for the enumeration of bacterial species in complex microbial systems [28]. Among the limitations, possible cross-reactions and varying reproducibility for different strains has been discussed [29]. In fact, the probes used were not particularly specific and did not differentiate between genotypes of the same species. For example, among some Gram-positive genera (e.g. Actinomyces, Lactobacillus and Streptococcus) there was a risk for cross-reactions between closely related species. Such cross-reactions between the probes were checked prior to the study and the selection of the present twelve DNA probes was done in order to get as few cross-reactions as possible. Furthermore, the selected species represented early colonizers of the oral cavity (mucosal membranes and teeth) and therefore considered suitable for this low age group. The methodological limitations made us however to present data according as proportions over the thresholds 10^4^ and 10^5^ cells respectively [29]. We used the swab-technique to collect oral samples rather than dental samples for practical reasons; because of the low age of the subjects, it was not possible to collect enough plaque and saliva for separate analysis. One should also keep in mind that the present assay only mirrored 12 selected species out of the over 600 prevalent taxa at species level that are reported from the oral cavity [30].

## Conclusions

Within the limitations of the present study, the findings suggested that the composition of the oral microflora did not differ in preschool children involved in a 2-year community fluoride varnish program when compared with those attending a standard oral health program without fluoride varnish applications.

## Abbreviations

CCUG: Culture Collection University of Gothenburg
ECC: Early childhood caries
ICDAS: International Caries Detection and Assessment System
OMGS: Oral microbiology Gothenburg Sweden
Ppm: Parts per million
SEK: Swedish Crowns
